# Quality of Life Analysis and Smoking Correlation in Symptomatic Spine Osteoarthritis: A Nationwide Health Survey Analysis of an Elderly Population with EQ-5D

**DOI:** 10.1371/journal.pone.0151315

**Published:** 2016-03-18

**Authors:** Jae-Young Hong, Kyungdo Han, Dong-Hyup Shin, Eun Mi Chun

**Affiliations:** 1 Department of Orthopedics, Korea University Ansan Hospital, Ansan, South Korea; 2 Department of Biostatistics, College of Medicine, Catholic University, Seoul, South Korea; 3 Division of Pulmonary and Critical Care Medicine, Department of Internal Medicine, Ewha Womans University Mokdong Hospital, Seoul, South Korea; University of Florida, UNITED STATES

## Abstract

**Objectives:**

To analyze quality of life in people with symptomatic spine osteoarthritis (OA) using the results of a cross-sectional, nationwide survey.

**Materials and Methods:**

This study used data from the Fifth Korean National Health and Nutrition Examination Survey (KNHANES V-5; 2010–2012). After excluding ineligible subjects, the total number of subjects in the study was 8,963, including 4,091 males and 4,872 females. All participants reported disabilities related to spine OA. Plain radiographs of the spine were taken for all participants.

**Results:**

Age, sex, smoking, drinking, education, and income level were significantly related to spine OA morbidity (P<0.05). OA morbidity was significantly higher in female ex-smokers (OR; 2.94, P<0.05). Quality of life (EQ-5D: L1~5) was significantly compromised in the group with spine OA compared to the group without spine OA (P<0.05). Overall, LQ 1, 2, 3, 4, and 5 domain scores were significantly higher in the group with spine OA (P<0.05). In the group with spine OA, quality of life was reduced on more than three questions for 34.3% of the group (EQ-5D: grade≥2); on two questions, for 18.5% of the group; and on one question, for 11.1% of the group. Mental stress, melancholy, and suicidal thinking were also more common in the group with spine OA (P<0.05). The group with radiographic spine OA but without symptoms did not have compromised EQ-5D scores, whereas the group with radiographic OA and symptoms showed a significantly reduced quality of life.

**Conclusions:**

Quality of life was significantly reduced in the group with symptomatic spine OA in a large cross-sectional analysis. Physicians should consider quality of life in the treatment of patients with spine OA

## Introduction

Spine osteoarthritis (OA) with or without nerve compression plays a significant role in the development of symptomatic spinal disease. Fifteen percent of the US population suffers from pain related to OA, and this figure is predicted to double by 2020.[1–3] Many OA patients also suffer from significant pain, disability, and reduced quality of life.[4–10] Consequently, health-related quality of life (HRQOL) is a key outcome in OA, but few population-based studies have examined the relationship between specific arthritic conditions, such as osteoarthritis (OA) and rheumatoid arthritis (RA), and HRQOL.[4, 7, 11, 12] To diagnose and treat degenerative spine OA, physicians must be familiar with the characteristics and prevalence of OA, as well as related disabilities in the development of OA. In this large, cross-sectional study, we assessed the disabilities associated with degenerative spine OA. We also considered whether smoking might attenuate the development of OA due to the protective effects of nicotine. The aim of this study was to investigate the relationship between quality of life and spine OA by using data from the Korean National Health and Nutrition Examination Survey (KNHANES).

## Materials and Methods

### Study Population

This cross-sectional study used data from the Fifth KNHANES-V: 2010–2012. The KNHANES is a nationwide health and nutrition survey conducted by the Korea Centers for Disease Control and Prevention. A stratified, multistage probability sampling design was used, and sampling units were based on geographical area, age, and sex.[6, 13, 14] The study subjects had undergone physical and laboratory examinations, including radiographic examination of the spine. In addition, health interview data were retrieved from the KNHANES, including demographic and lifestyle variables (physical activity and mental status). All subjects provided written informed consent, and the Institutional Review Board of the Korea Centers for Disease Control and Prevention approved the study protocol.

### Radiographic Examination

Spine radiography was performed with a SD3000 Synchro Stand (Accele Ray, Switzerland). Anteroposterior and lateral plain radiographs of the spine were taken. Radiographic changes in each joint were then independently assessed by two radiologists using the Kellgren/Lawrence (KL) grading system: Grade 0, visible features of OA are absent or doubtful, such as questionable osteophytes; Grade 1, minimal, with definitive small osteophytes; Grade 2, definitive moderate osteophytes or subchondral bone cysts and sclerosis with or without foraminal stenosis.[15] The presence of radiographic OA was defined as a KL grade greater than or equal to 2. If the grades given by the two radiologists differed by 1 KL grade for the same case, the higher grade was accepted.[16] In addition, all subjects described their current symptoms related to spine OA, and the symptoms were scored. Subjects who had experienced arthritic pain for more than 30 days in the past three months were asked to report the average intensity of pain using an 11–point numeric rating scale ranging from 0 to 10 (higher values indicate higher pain).[14]

### Demographic and Lifestyle Variables

Demographic variables included age, sex, income, marital status, current residence, education level, smoking status, alcohol consumption, and physical activity. Equivalized household income was calculated as the total monthly household income divided by the square root of the total number of household members. Average alcoholic beverage consumption was assessed with a self-reported questionnaire and then converted into the amount of pure alcohol consumed per day. Education level was classified as low, intermediate and high (<middle school, high school, >college). Subjects who had smoked more than 100 cigarettes in their lives were classified as ever-smokers. Smoking status was further classified as non-, ex- and current smokers. Physical activity was assessed with the Korean version of the International Physical Activity Questionnaire short form.[14]

### Anthropometric and Laboratory Measurements

Body weight and height were measured, and body mass index (kg/m^2^) was calculated. Waist circumference was measured at the narrowest point, between the lower costal margin and the iliac crest. Systolic blood pressure and diastolic blood pressure were measured with a standard mercury sphygmomanometer (Baumanometer; Baum, Copiague, NY, USA). Hypertension was defined as blood pressure greater than 140/90 mmHg, or current use of antihypertensive medications.[14]

### Quality of Life Variables

HRQOL was evaluated with the EQ-5D, a self-reported questionnaire widely used to assess the quality of life of the general population (Table 1).[17–20] The Korean EQ-5D was developed according to guidelines from the EQ group, and its reliability and validity have been tested on patients with rheumatism, cancer, and chronic disease.[13] The EQ-5D consists of five questions evaluating a respondent’s current health status in terms of mobility, self-care, usual activities, pain/discomfort, and anxiety/depression. For each dimension, a respondent can belong to 1 of 3 categories, including “no problems,” “moderate problems,” and “severe problems.”

**Table 1:** EQ-5D questions with specific domain (Grade 1~3).

**LQ-1: Have disability to exercise (EQ-5D)**
**G1. I have no problems in walking about**
**G2. I have some problems in walking about**
**G3. I am confined to bed**
**LQ-2: Have disability to self-care (EQ-5D)**
**G1. I have no problems with self-care**
**G2. I have some problems washing or dressing myself**
**G3. I am unable to wash or dress myself**
**LQ-3: Have disability to daily living (EQ-5D)**
**G1. I have no problems with performing my usual activities**
**G2. I have some problems with performing my usual activities**
**G3. I am unable to perform my usual activities**
**LQ-4: Have pain (EQ-5D)**
**G1. I have no pain or discomfort**
**G2. I have moderate pain or discomfort**
**G3. I have extreme pain or discomfort**
**LQ-5: Have depression or anxiety (EQ-5D)**
**G1. I am not anxious or depressed**
**G2. I am moderately anxious or depressed**
**G3. I am extremely anxious or depressed**

### Statistical Analysis

Statistical analyses were conducted by using SAS survey procedures (version 9.3; SAS Institute, Cary, NC, USA) in a manner that reflected sampling weights and provided nationally representative estimates. The characteristics of the subjects with spine OA were compared with those of subjects without spine OA (control group) using independent sample *t*-tests and a one-way analysis of variance for continuous variables, and chi-squared tests for categorical variables. Multivariable logistic regression analyses were conducted to investigate the relationships between parameters. SES variables with a P-value less than 0.15 in analyses were selected for multivariable analyses. P-values less than 0.05 were considered statistically significant.[21]

## Results

**Fig 1:**
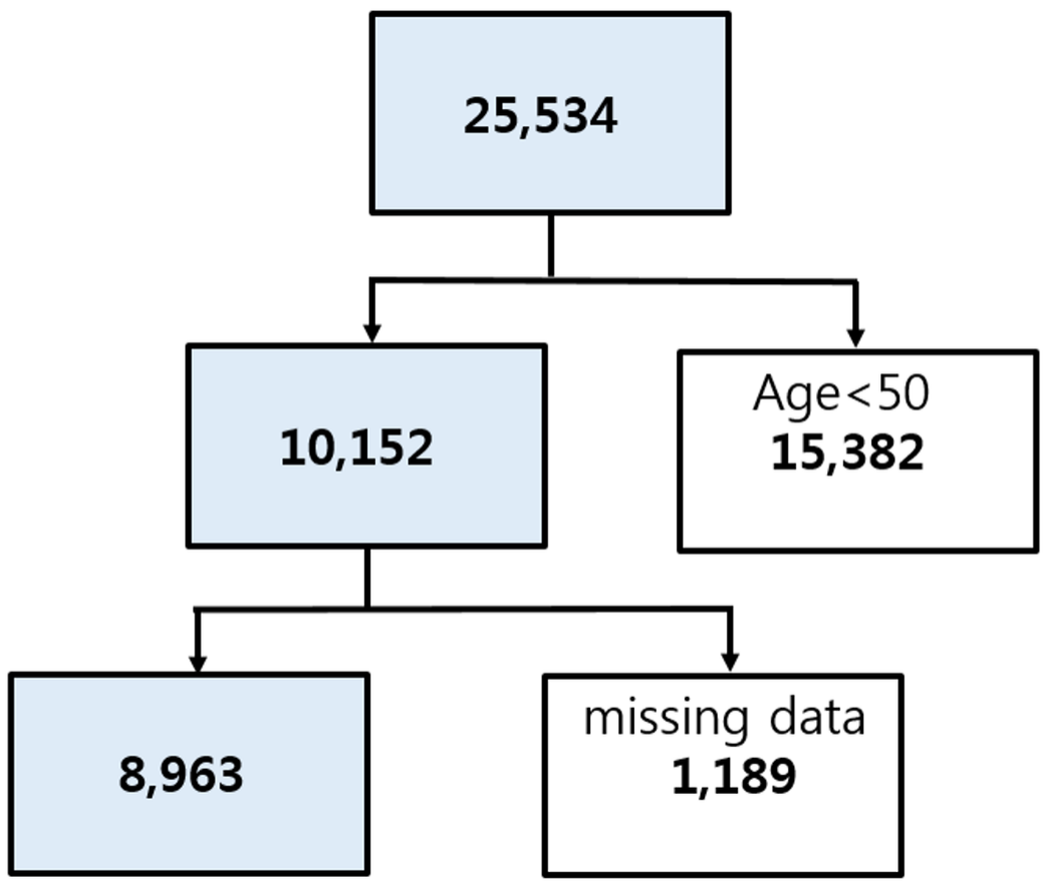
Flow chart showing inclusion and exclusion of subjects in relation to the study criteria.

There were 25,534 subjects included in the health interview survey and health examination survey (Fig 1). We excluded persons younger than 50 years of age (n = 15,382) and those missing data for our variables of interest (n = 1,189). After ineligible subjects were excluded, the total number of subjects in the study was 8,963, including 4,091 males and 4,872 females.

### Factors Related to Spine OA

We defined spine OA as “definite osteoarthritis on plain radiographs with related pain of the spine.” Age, sex, smoking, and drinking were significantly related to spine OA morbidity (Table 2). Only female ex-smokers were significantly associated with spine OA morbidity (odd ratio: 2.94, Table 3). Education and income were significantly related to spine OA morbidity. Likewise, HTN and metabolic syndrome were significantly related to OA (P<0.05). Significance levels were higher for these factors in females with a higher prevalence of spine OA (P<0.05).

**Table 2:** Comparison of prevalence of risk factors between spine OA and control group.

Spine OA	without	with	
n	7834	1129	p
**Age>65**	61.3%(0.2)	69.9%(0.4)	<0.0001
**Sex (female)**	51.1%(0.5)	77.0%(1.5)	<0.0001
**Smoke**	18.6%(0.6)	12.4%(1.3)	0.0002
**Heavy drink**	49.1%(0.7)	28.3%(1.7)	<0.0001
**Heavy exercise**	17.9%(0.6)	18.2%(1.8)	0.8224
**With spouse**	81.7%(0.6)	57.8%(1.9)	<0.0001
**High education**	37.8%(0.9)	12.3%(1.4)	<0.0001
**Low Income**	25.8%(0.9)	52.6%(2.0)	<0.0001
**Mental stress**	21.2%(0.5)	32.4%(1.6)	<0.0001
**DM**	16.6%(0.5)	19.9%(1.8)	0.0532
**HTN**	48.0%(0.8)	61.0%(1.9)	<0.0001
**MS**	43.5%(0.7)	51.9%(2.1)	<0.0001

All the values are adjusted for statistical comparisons.

(P<0.05- statistical significance; (): standard error)

DM: diabetes mellitus; HTN: hypertension; MS: metabolic syndrome

**Table 3:** Prevalence and risks of smoking with spine osteoarthritis.

	Male		Female		Total	
	Prevalence	Odd	Prevalence	Odd	Prevalence	Odd
**Non-**	4.6%(1.1)	1	15.4%(0.7)	1	14.0%(0.6)	1
**Ex-**	5.7%(1.1)	1.17(0.61–2.23)	35.2%(8.1)	2.94(1.27–6.84)	17.8%(1.3)	1.43(0.91–2.25)
**Current**	6.4%(0.8)	1.38(0.78–2.47)	17.0%(3.2)	1.12(0.67–1.87)	17.8%(0.9)	1.34(0.94–1.90)
P	0.438	0.523	0.006	0.039	<0.0001	0.173

All the values are adjusted for statistical comparisons.

(P<0.05- statistical significance; (): standard error)

Age, BMI, drink, exercise, DM, HTN, MS and stress were adjusted for statistical analysis.

Non-: Non-smoker; Ex-: Ex-smoker, Current: current smoker.

Confidence intervals are reported in parentheses (OR).

### Quality of Life and Disabilities with Spine OA

EQ-5D and EQ VAS scores were significantly compromised in the spine OA group. LQ 1, 2, 3, 4, and 5 scores were significantly higher in the spine OA group (Table 4, P<0.05). Overall, quality of life was compromised on more than three questions in 34.3% of the spine OA group (grade ≥2); on two questions in 18.5% of the spine OA group; and on one question, in 11.1% of the spine OA group. The group that had radiographic spine OA without symptoms did not show compromised EQ-5D scores, but the group that had radiographic OA plus symptoms showed a significantly compromised qualify of life (Table 5).

**Table 4:** Comparison of EQ-5D result between spine OA and control group.

Spine OA	Without	With	p
**Mobility**			<0.0001
G1	78.1%(0.6)	32.9%(1.7)	
G2	21.1%(0.6)	62.0%(1.8)	
G3	0.8%(0.1)	5.1%(0.8)	
**Self-care**			<0.0001
G1	94.2%(0.3)	74.3%(1.6)	
G2	5.6%(0.3)	23.4%(1.6)	
G3	0.3%(0.1)	2.3%(0.6)	
**Usual activities**			<0.0001
G1	87.6%(0.5)	50.8%(1.9)	
G2	11.2%(0.5)	40.7%(1.9)	
G3	1.2%(0.2)	8.5%(1.0)	
**Pain/discomfort**			<0.0001
G1	73.5%(0.6)	31.2%(1.7)	
G2	23.4%(0.6)	52.3%(1.8)	
G3	3.1%(0.3)	16.5%(1.5)	
**Anxiety/discomfort**			<0.0001
G1	86.9%(0.5)	72.9%(1.6)	
G2	12.2%(0.5)	23.8%(1.5)	
G3	1.0%(0.1)	3.3%(0.6)	
**EQ5D**	0.923±0.002	0.754±0.008	<0.0001
**EQ_VAS**	78.3±1.2	81.7±7.6	<0.0001

All the values are adjusted for statistical comparisons.

(P<0.05- statistical significance; (): standard error)

**Table 5:** Calculated risks of compromised EQ-5D in spine OA group.

	Mobility	Self-care	Usual activities	Pain/Discomfort	Anxiety/Depression
**Prevalence (G 2&3)**					
**OA+Sx**	67.1%(1.7)	25.7%(1.6)	49.2%(1.9)	68.8%(1.7)	27.1%(1.6)
**Sx**	46.2%(1.9)	14.2%(1.2)	32.0%(1.8)	60.1%(2.0)	27.6%(1.8)
**OA**	28.9%(1.2)	6.5%(0.7)	13.9%(0.9)	26.6%(1.2)	10.8%(0.8)
**Control**	14.2%(0.6)	3.7%(0.3)	7.6%(0.5)	19.0%(0.7)	10.7%(0.6)
**P-value**	<0.0001	<0.0001	<0.0001	<0.0001	<0.0001
**Odd ratio**					
**OA+Sx**	6.19(4.99–7.69)	4.34(3.14–5.99)	5.97(4.69–7.61)	6.32(5.19–7.69)	2.12(1.65–2.71)
**Sx**	4.20(3.44–5.12)	3.00(2.26–3.99)	4.44(3.53–5.59)	5.34(4.40–6.47)	2.45(1.95–3.08)
**OA**	1.59(1.33–1.91)	1.12(0.77–1.62)	1.26(1.00–1.59)	1.34(1.13–1.59)	0.90(0.72–1.14)
**Control**	1	1	1	1	1

All the values are adjusted for statistical comparisons.

(P<0.05- statistical significance; (): standard error)

OA: spine osteoarthritis; Sx: symptom.

Confidence intervals are reported in parentheses (OR).

#### EQ-5D mobility

The majority of the spine OA group had grade 2 disability, whereas the control group had grade 1. The calculated risk for compromised mobility was 6.19 (odd ratio) compared to the control group.

#### EQ-5D self-care

The majority of the spine OA and control groups had grade 1 disability. However, the incidence of grade 2 or 3 disability was higher in the spine OA group. In particular, grade 3 disability was higher than other domains (2.3%) in the spine OA group. The calculated risk for compromised mobility was 4.34 compared to the control group.

#### EQ-5D usual activities

The majority of the spine OA and control groups had grade 1 disability. However, the incidence of grade 2 or 3 disability was higher in the spine OA group. The calculated risk for compromised mobility was 5.97 compared to the control group.

#### EQ-5D pain/discomfort

The majority of the spine OA group had grade 2 disability, whereas the control group had grade 1. In particular, grade 3 disability was higher than other domains (16.5%) in the OA group. The calculated risk for compromised mobility was 6.32 compared to the control group.

#### EQ-5D anxiety/discomfort

The majority of the spine OA and control groups had grade 1 disability. However, the incidence of grade 2 or 3 disability was higher in the spine OA group. The calculated risk for compromised mobility was 2.12 compared to the control group.

## Discussion

Many physicians focus on the treatment of spinal disease, but spine OA itself can induce disability. Although we focused on stenosis and listhesis specifically, OA itself should be considered as a specific modality. Spine OA, a chronic condition of the elderly population, is related to several chronic diseases and socio-economic problems. In this study, we found that the group with spine OA showed a higher prevalence of being single, low income, and low education, which indicates that a compromised social life may increase the incidence of spine OA or vice versa. In addition, the group with spine OA was more likely to have DM, HTN, and metabolic syndrome than the group without spine OA, as reported in previous studies.[1, 22–29] The prevalence of drinking and smoking was unexpectedly higher in the control group. It appears that the female dominance of the spine OA population showed adverse results. The components of tobacco smoke increase the risk of developing back pain because of their negative effects on chondrocyte function in the disc.[30, 31] Some studies also report that patients with OA who smoke have greater cartilage loss and more severe knee joint pain.[32, 33] In contrast, smoking has been reported to have some protective association with OA.[34, 35] The effects of nicotine on osteoblast formation and function appear to be dose-dependent.[36] Hui et al. reported that of 48 studies (537,730 participants), a negative association appeared only in hospital settings (odd ratio: 0.65), and they concluded that the protective effects of smoking in OA may be caused by selection bias in a hospital setting.[37] Therefore, we evaluated the association between smoking and OA in the present study. Among females, OA was more common among ex-smokers than patients who never smoked, and current smokers also showed a significantly higher incidence of OA(P<0.05). These results suggest that the components of tobacco smoke have a harmful effect on chondrocyte function and increase the prevalence of OA. Nicotine alone may have some inverse association with OA, but tobacco has many detrimental effects on the musculoskeletal system.

The group with spine OA showed many related risk factors and a higher incidence of other diseases, indicating that the impact of spine OA itself should be studied. However, physicians have tended to focus on the related medical problems and risk factors of spine OA, with few reports analyzing the quality of life or mental status of patients with spine OA. Geryk et al. reported that arthritis negatively impacts HRQOL; patients with OA perceived their condition as disabling, similar to that of patients with RA. They also reported that OA patients had poor physical and mental health.[11] In the present study, we hypothesized that quality of life in the group with spine OA affects patients’ overall health status, a topic that has not been studied to date. Therefore, we evaluated the relationship between symptomatic spine OA and quality of life to determine the extent to which quality of life was compromised by spine OA. Dominick et al. reported that OA has a significant impact on multiple dimensions of HRQOL among older adults.[7] They reported that subjects with OA and RA had worse scores than those without arthritis on all HRQOL items, including general health, physical health, mental health, activity limitations, pain, and sleep. In the present study, we found a significant reduction in quality of life among patients with spine OA. The EQ-5D questions revealed that patients with spine OA had significant pain and discomfort, and their mobility and usual activities were also significantly compromised. Thus, it appears that symptomatic spine OA restricts usual activities and mobility due to significant pain. Although the anxiety and depression domain was also compromised in the group with spine OA, it showed the smallest odds ratio when compared to the control group, which indicates that spine OA had less of an effect on mental status.

There are several limitations to the present study. The cross-sectional study design prevented us from determining an exact cause-and-effect relationship. Future prospective studies are required to better elucidate causal relationships. Furthermore, longitudinal studies are also necessary in order to take (time-constant) unobserved heterogeneity into account as discussed in AgeCoDe studies.[38, 39] In addition, more sophisticated diagnostic tools (MRI, CT, etc.) are needed to evaluate the precise status of joints. In this study, we found a mismatch between radiographic OA and symptoms or disabilities. Radiographic OA does not always indicate symptomatic disease with disability. We could not find any significant compromise in quality of life in the group with radiographic spine OA, which emphasizes the importance of identifying clinical symptoms in the treatment of spine OA. There are many cases of asymptomatic radiographic spine OA and vice versa; therefore, physicians should double-check for symptoms and radiographic abnormalities. In addition, it is very difficult to generalize our results to other populations because we focused on Korea, and other countries may be different due to ethnic differences. Despite these limitations, we found a significant reduction in quality of life among patients with spine OA in a large cross-sectional population, and the degree to which quality of life was reduced was higher than expected. We believe that our results will be helpful to physicians in the treatment of spine OA. Due to the rising number of older adults in many countries, the public health burden of spine OA is expected to increase dramatically. Efforts are needed to enhance access to medical care and disseminate self-management interventions for arthritis. Thus, physicians should consider quality of life in the treatment of spine OA.

## Conclusion

In the present study, quality of life was significantly compromised in the population with symptomatic spine OA. LQ 1, 2, 3, 4, and 5 domain scores were significantly higher in the group with spine OA. Physicians should consider quality of life in the treatment of patients with spine OA. In addition, female ex-smokers had a high prevalence of OA, and smoking cessation appears to be an important prevention strategy for OA.

## Statement of Ethics

All subjects provided written informed consent, and the Institutional Review Board of the Korea Centers for Disease Control and Prevention approved the study protocol (IRB No 2010-02CON-21-C, 2011-02CON-06-C, 2012-01EXP-01-2C).
